# Insulin Stimulates Translocation of Human GLUT4 to the Membrane in Fat Bodies of Transgenic *Drosophila melanogaster*

**DOI:** 10.1371/journal.pone.0077953

**Published:** 2013-11-06

**Authors:** Georgeta Crivat, Vladimir A. Lizunov, Caroline R. Li, Karin G. Stenkula, Joshua Zimmerberg, Samuel W. Cushman, Leslie Pick

**Affiliations:** 1 Department of Entomology, University of Maryland, College Park, Maryland, United States of America; 2 Program in Physical Biology, Eunice Kennedy Shriver National Institute of Child Health and Human Development, National Institutes of Health, Bethesda, Maryland, United States of America; 3 Experimental Diabetes, Metabolism, and Nutrition Section, Diabetes, Endocrinology, and Obesity Branch, National Institute of Diabetes and Digestive and Kidney Diseases, National Institutes of Health, Bethesda, Maryland, United States of America; University of Massachusetts Medical School, United States of America

## Abstract

The fruit fly *Drosophila melanogaster* is an excellent model system for studies of genes controlling development and disease. However, its applicability to physiological systems is less clear because of metabolic differences between insects and mammals. Insulin signaling has been studied in mammals because of relevance to diabetes and other diseases but there are many parallels between mammalian and insect pathways. For example, deletion of *Drosophila* Insulin-Like Peptides resulted in ‘diabetic’ flies with elevated circulating sugar levels. Whether this situation reflects failure of sugar uptake into peripheral tissues as seen in mammals is unclear and depends upon whether flies harbor the machinery to mount mammalian-like insulin-dependent sugar uptake responses. Here we asked whether *Drosophila* fat cells are competent to respond to insulin with mammalian-like regulated trafficking of sugar transporters. Transgenic *Drosophila* expressing human glucose transporter-4 (GLUT4), the sugar transporter expressed primarily in insulin-responsive tissues, were generated. After expression in fat bodies, GLUT4 intracellular trafficking and localization were monitored by confocal and total internal reflection fluorescence microscopy (TIRFM). We found that fat body cells responded to insulin with increased GLUT4 trafficking and translocation to the plasma membrane. While the amplitude of these responses was relatively weak in animals reared on a standard diet, it was greatly enhanced in animals reared on sugar-restricted diets, suggesting that flies fed standard diets are insulin resistant. Our findings demonstrate that flies are competent to mobilize translocation of sugar transporters to the cell surface in response to insulin. They suggest that *Drosophila* fat cells are primed for a response to insulin and that these pathways are down-regulated when animals are exposed to constant, high levels of sugar. Finally, these studies are the first to use TIRFM to monitor insulin-signaling pathways in *Drosophila*, demonstrating the utility of TIRFM of tagged sugar transporters to monitor signaling pathways in insects.

## Introduction

Insulin regulation of sugar homeostasis has been extensively investigated because of its importance in diabetes and related syndromes [1]. In humans and other mammals, glucose is transported from circulation into adipose and muscle tissues by insulin-dependent glucose transporter proteins (reviewed in [1]–[7]). GLUT4 is the key insulin-dependent sugar transporter: in response to insulin binding to its receptor (IR), a downstream cascade is initiated which results in the redistribution of GLUT4 from intracellular stores to the plasma membrane, allowing facilitated glucose uptake from the circulation [2], [6], [7]. Total Internal Reflection Fluorescence Microscopy (TIRFM) of GLUT4 trafficking in mammalian white adipose cells showed that GLUT4 resides in vesicles that move rapidly along a microtubule network. Insulin stimulation decreases trafficking due to tethering and fusion of GLUT4 vesicles with the plasma membrane [5], [8], [9].

Although invertebrates differ greatly in many aspects of their physiology from mammals, they provide the opportunity to model human disease because they are easily reared in large numbers and powerful genetic tools are available. An insulin-like signaling system has been well-characterized in the fruit fly, *Drosophila melanogaster* [10]. The *Drosophila* insulin receptor (DInR) is similar in sequence to mammalian insulin receptor and auto-phosphorylates in response to mammalian insulin [11], [12]. DInR controls growth, metabolism and other processes in the fly and many components of mammalian IR signaling cascades have been identified in flies. For example, an IRS-like adapter protein, Chico, homologous to vertebrate insulin receptor substrates (IRS1-4), interacts with DInR to regulate cell and organismal growth [13], via a highly conserved downstream signaling pathway [12]–[18]. Similarly, an evolutionarily conserved SH2B-family adaptor protein Lnk regulates cellular and organismal growth [19]. In mammalian cells, insulin stimulation triggers a cascade of phosphorylation events, which, in addition to other outcomes, results in the activation of PI3K which further phosphorylates inositol lipids (e.g., phosphatidylinositol-4,5-bisphosphate generating PIP_3_, PtdIns (3,4,5)P3 [20]. There is also a PI3K independent pathway which involves production of PtdIns-3-P (Phosphatidylinositol-3-phosphate) through the GTP-binding protein TC10, in response to insulin stimulation. [21]. The presence of these lipid signals in mammalian adipose cells was shown to be important for insulin-induced GLUT4 translocation to the membrane and sugar uptake [21], [22]. In *Drosophila*, as in mammalian cells, insulin stimulation results in activation of PI3K pathways and generation of PtdIns(3,4,5)P3 in the plasma membrane, followed by activation of protein kinase PKB/AKT [14], [23]. In contrast, nutritional status and not insulin controls the presence of PtdIns-3 in the membrane [14], [24]. Thus, despite some differences, intracellular insulin-signaling pathways appear to be largely conserved between mammals and insects.

There is ample data in the insect literature documenting alternate mechanisms of sugar metabolism and utlization in insects. While mammals suffer both short and long term consequences from high levels of circulating glucose, insects have adaptations that involve utilization of high sugar levels as a means of survival. The primary circulating sugar in insects is trehalose [25], a nonreducing disaccharide that is also the primary circulating sugar elevated in *dilp* mutant flies [26]. Many insects raise trehalose levels seasonally, as trehalose functions as a cryoprotectant. Trehalose also provides resistance to dehydration and heat stress (reviewed in [27]–[29]. In fact, a number of physiological studies of honeybees and other insects suggest an absence of homeostatic regulation of sugar metabolism, as insects appear to be primed to raise sugar levels in response to high activity levels required for flight, while having no necessity to lower levels, since trehalose is a neutral sugar [30]. For example, using direct measurements of radiolabelled compounds, Thompson et al. found that hemolymph trehalose levels increased with increasing sugar uptake in *Manduca sexta* and that this trehalose was synthesized directly from dietary intake [31]. Further, they found that injection of glucose did not result in down regulation of trehalose synthesis [32]. Further arguing against hormonal regulation they found that levels of circulating sugar were not maintained upon starvation, but rather decrease dramatically in several insect species [31], [33]. Three genes encoding candidate sugar transporters have been annotated in the *Drosophila* genome, but little is known about their function (see also discussion). In light of the physiological differences summarized above, we questioned the extent to which insulin regulation of sugar homeostasis is shared between flies and mammals.

As a first step to address this, human GLUT4, the primary sugar transporter in insulin-responsive tissues, was expressed in the fat bodies of transgenic *Drosophila*. GLUT4 trafficking and subcellular localization were monitored by confocal and TIRFM in fat bodies of animals fed on standard or sugar-restricted diets. We found that *Drosophila* fat cells responded to insulin by increasing GLUT4 trafficking and translocation to the membrane, with responses much stronger in sugar-restricted animals. These studies support the notion that insects hormonally regulate sugar homeostasis and that they have the machinery to mobilize translocation of sugar-transporters to the membrane in response to insulin.

## Materials and Methods

### Generation of Transgenic Flies

A ∼2.4 kb fragment carrying *HA-GLUT4-GFP* was excised from *HA-GLUT4-GFPpQB125* [8] using *SacII* and *XbaI* and inserted into *SacII* and *XbaI* sites of the P-element vector *pUAST* to generate *UAS-HA-GLUT4-GFP.* Transgenic flies were generated by Rainbow Transgenic Flies (Camarillo, CA). Multiple independent lines were established. To determine expression levels, transgenic *UAS-HA-GLUT4-GFP/UAS-HA-GLUT4-GFP* males were crossed to virgin females carrying the fat body *GAL4* driver *P{GawB}c564* [34] (Bloomington Number *6982*). Fat body expression of GFP was analyzed by fluorescent microscopy and lines expressing intermediate levels of GFP were chosen for further experiments. These levels allowed for clear detection of GFP, but avoided issues of over-expression that have been shown to interfere with trafficking [35]. For all experiments shown, ten virgin females homozygous for *P{GawB}c564* were crossed to ten *UAS-HA-GLUT4-GFP/UAS-HA-GLUT4-GFP* males. Experiments were repeated with two independent transformant lines that express GFP at intermediate levels (10C1 and 78A3; Fig. S1). To test insulin-responsiveness of HA-GLUT4-GFP, fat bodies from wandering third instar larvae were dissected, incubated with 0.1 U/ml human insulin (Invitrogen) for 5–30 minutes, and imaging was performed by confocal or TIRF microscopy, as described in more detail below.

### Fly Maintenance and Diet

Flies were maintained in a 25°C incubator on a standard cornmeal diet containing: 54 g yeast (Red Star Active Dry Yeast), 45 ml molasses, 5 g agar, 4 ml propionic acid, 11 ml of 10% tegosept dissolved in 100% ethanol and 94 g cornmeal, per 1 liter in distilled water. Standard food contains molasses (Golden A) (4.6%), yeast (5.5%), and cornmeal (9.5%) as nutritionally active ingredients. Molasses contains 14 g total sugar and 16 g total carbohydrates. Sugar-restricted food contained all components of standard food with the exception that molasses, the major source of sugar, was not added. Note that although the food wasn’t enriched with sugar it was not sugar-free as the cornmeal contains 2% sugars and 31% total carbohydrates whereas yeast contains no sugar but ∼37% total carbohydrates. Third instar larvae were viable and grew at normal rates on this food source. Moreover, approximately 50% of these developed into viable adults. For the starvation experiments, *Drosophila* larvae were grown on standard food containing 0.05% FD&C blue dye [36]. For starvation experiments early third instar larvae (identified by their blue guts) were extracted from food, washed and grown for 24 h on watered cotton swabs.

### Immunostaining and Confocal Microscopy

Wandering transgenic *P{GawB}c564>HA-GLUT4-GFP* third instar larvae were dissected in chilled PBS solution. Fat bodies were isolated and transferred to Krebs-Ringer bicarbonate/Hepes buffer containing 1% BSA (KRBH) solution for physiological studies of insulin stimulation [37]. For imaging of surface exposed GLUT4, non-permeabilized tissue from freshly harvested fat bodies was adsorbed on a 0.2 µm filter membrane (Millipore) and placed into Glass Bottom Culture Dishes (MatTek). Isolated tissue was either kept in KRBH (basal) or 0.1 U/ml human insulin (I2643, Sigma) was added. Tissue was incubated for 30 min at room temperature (RT). The tissue was then washed twice with PBS and fixed with 4% paraformaldehyde in PBS for 10 min at RT. Mouse monoclonal anti-HA antibody (Covance) was added at a 1∶500 dilution in KRBH and incubated for 20 min at RT. Then tissue was washed three times with KRBH and incubated with secondary goat anti-mouse Alexa 647 antibody (Invitrogen) at a dilution of 1∶500 for 20 min at RT. Samples were washed three times with KRBH and 5 µM Hoechst was added. For testing effects of wortmannin on HA-GLUT4-GFP translocation, fat bodies from animals fed on a sugar-restricted diet were isolated and pre-incubated in RPMI for 20 min with 100 nM/L wortmannin (Enzo Life Sciences Inc.) at RT prior to insulin addition.

For imaging of intracellular GLUT4, isolated and fixed fat bodies were permeabilized with 0.5% saponin in KRBH, and incubated with rabbit polyclonal anti-GLUT4 antibody [38] at a dilution of 1∶1000 for 20 min at RT. Following the incubation, the samples were washed three times with KRBH containing 0.5% saponin and secondary antibody (goat anti-rabbit Alexa 647) was added at a dilution of 1∶1000 followed by incubation for 20 min at RT. Unbound antibodies were washed three times with KRBH containing 0.5% saponin.

Tissue samples were transferred to an LSM510 confocal microscope (Carl Zeiss Micro Imaging) and imaged with a planapochromat 60×1.4 NA oil-immersion objective. Confocal images were obtained using multi-track excitation with 405 nm (Hoechst), 488 nm (GFP) and 633 nm (Alexa 647) lasers. Five to ten confocal sections were collected for each cross-sectional field of view of fat body tissue with cell plasma membranes perpendicular to the focal plane. Tissue samples from at least three individual larvae were recorded for each experimental condition.

### TIRF Microscopy of Isolated Fat Body Tissue

Total Internal Reflection Fluorescence Microscopy (TIRFM) was used to examine GLUT4 trafficking in live cells of isolated fat bodies. Objective-based TIRFM setup was built around a Nikon Eclipse Ti microscope equipped a TIRF-illumination arm, custom-built laser combiner, and an Ixon EMCCD camera (Andor). A laser combiner operated four diode-pumped solid-state lasers (405, 488, 561 and 640 nm, Coherent) controlled via Uniblitz shutters (Vincent Associates). A 60×1.49 NA TIRF lens was used for through-the-objective TIRF and wide-field illumination modes. The incident angle of the laser beam was controlled by a motorized TIRF-unit and switched between pre-calibrated settings corresponding to TIRF, and wide-field illumination. Penetration depth of the evanescent field was measured to be 110±10 nm by a calibration procedure with 40 nm fluorescent beads attached to the piezo-driven micropipette.

Fluorescence signals generated during acquisition were separated from the excitation light using quad-band dichroic and emission filter set (405/488/561/640, Semrock). An additional filter-wheel (Lambda 10B, Sutter) was used to select between green and red emission bands using appropriate filters (535/30 nm and 609/50 nm, Semrock). Laser-combiner, shutters, filter-wheels, microscope, and EMCCD camera were synchronized and controlled using Micro-Manager 1.3 [39]. Nikon “perfect focus” was activated throughout the time-lapse recordings to avoid focus drift.

A series of 240 time lapse images were obtained at the rate of 1 fps under basal conditions and after 5, 10 and 15 min after insulin addition. GLUT4 trafficking was quantified as the number of mobile GLUT4-GFP particles detected in the vicinity of the plasma membrane per unit area during one min intervals. Stacks of 60 images representing 1 min of recording were processed to create maximum and mean projection image and were pixel by pixel subtracted. The resulting image contained the short-range trajectories of individual mobile particles. The number of trajectories were counted for 4 regions (10×10 µm) for each cell and represented as mean ± S.D. At least 3 cells were analyzed for each experimental condition.

### Statistical Analysis

Microsoft Excel was used to calculate data as Mean ± SD or Mean ± SEM. The statistical T test was used to compare between two groups (basal/insulin; basal/wortmannin+insulin; wortmannin+insulin/insulin). P values were calculated using Microsoft Excel type 1 T-test, paired for the wortmannin experiment, and for analysis of trafficking, type 3 unequal variances, unpaired T-test was used.

## Results

### Expression and Subcellular Localization of Human HA-GLUT4-GFP in Drosophila Fat

**Figure 1:**
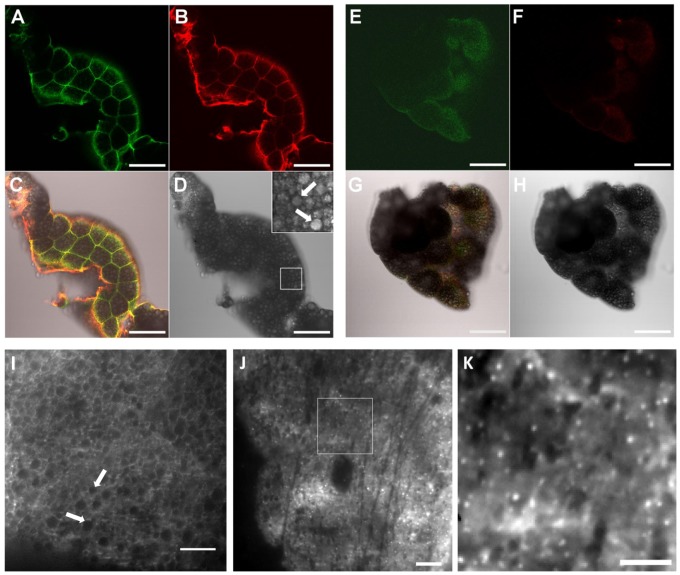
Expression and localization of HA-GLUT4-GFP in *Drosophila* fat. Confocal imaging of fat bodies from animals expressing: (A–D) *HA-GLUT4-GFP*; (E–H) negative control, *UAS-lacZ*. (A, E) GFP fluorescence (green); (B,F) Immunostaining with anti-GLUT4 (red); (C,G) Merge of images shows overlap of GFP and GLUT4 in transgenic fat (A–C) and little background in controls (E–G). (D, H) DIC images show abundant lipid droplets within each cell. (Panel D inset), enlarged image of fat body cells with multiple whitish lipid droplets (white arrows). Scale bars, 100 µm. (I–K) High resolution imaging of HA-GLUT4-GFP by TIRFM. Live fat body tissue was isolated and distribution of HA-GLUT4-GFP monitored using 488 nm laser illumination in TIRF mode. Scale bar, 5 µm. Note network of GLUT4 surrounding lipid droplets (I, white arrows) and presence of GLUT4 in punctate structures. Fig. 1J, representative projection image obtained by averaging 60 time frames from TIRF recordings, showing GLUT4 localized at the membrane. Scale bar, 10 µm. (K) Magnification of boxed region from (J) shows at a higher resolution, vesicular distribution of GLUT4, as well as GLUT4 associated with the plasma membrane. Scale bar, 5 µm.

We generated transgenic *Drosophila* carrying a *UAS-HA-GLUT4-GFP* transgene to express a doubly-tagged GLUT4 protein: an HA tag inserted in the first exofacial loop of GLUT4 to monitor expression at the cell surface [37] and C-terminal GFP to monitor transgene expression. Fat body tissue from transgenic third instar *Drosophila* larvae carrying a fat body *GAL4* driver, *P{GawB}c564*, and *UAS-HA-GLUT4-GFP* (Fig. 1A–D) or *UAS-lacZ* (Fig. 1E–H) was examined. Expression was monitored by GFP fluorescence (Fig. 1 A, E, green) or with anti-GLUT4 antibody (Fig. 1B, F, red) in permeabilized tissue samples. HA-GLUT4-GFP was clearly observed with complete co-localization of GFP fluorescence and anti-GLUT4 antibody staining, especially at the edges of the tissue samples where antibody penetrability was highest (Fig. 1C).

Expression of the *UAS-HA-GLUT4-GFP* transgene was confirmed in multiple independent transformant lines (Figs. 1A, S1 and data not shown). Background fluorescence was extremely low, as seen in *UAS-lacZ* controls (Fig. 1E–H), allowing us to use GFP to monitor gene expression in the fat bodies of *UAS-HA-GLUT4-GFP* transgenic animals.

Differential interference contrast microscopy (DIC) images of larval fat body tissue showed multiple lipid droplets packed within every cell (Fig. 1D, arrows in inset) similar to 3T3-L1 adipocytes and brown adipose cells [40]. Using TIRFM with an evanescent field depth of ∼100 nm, HA-GLUT4-GFP was detected in the vicinity of the plasma membrane primarily as bright punctate structures (Fig. 1J, K), similar to the localization of GLUT4 in vesicles in mammalian cells [8], [40]. Using TIRFM with increased penetration depth, HA-GLUT4-GFP was also detected in a net-like organization throughout the cytoplasm surrounding lipid droplets (Fig. 1I: GFP, white; lipid droplets, black regions). In sum, HA-GLUT4-GFP was effectively expressed and detected in the fat bodies of transgenic *Drosophila*. GLUT4 appears to be present in vesicles, although future work will be required to determine whether these correspond to GLUT4 Storage Vesicles (GSV) seen in mammalian cells (see Discussion).

### Larvae Reared on Sugar-restricted Diets show Strong GLUT4 Trafficking Responses to Insulin

**Figure 2:**
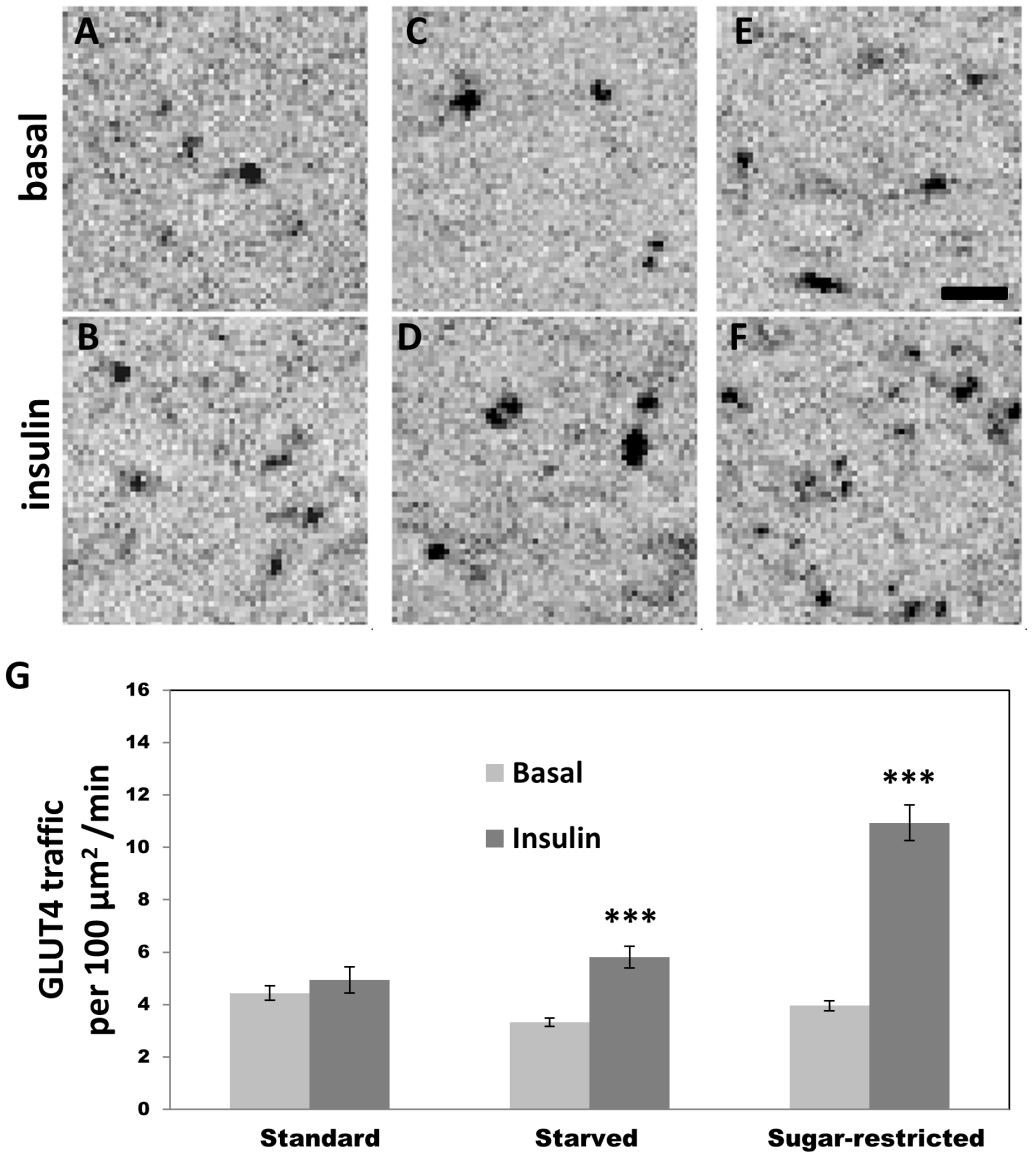
Insulin stimulates GLUT4 trafficking in *Drosophila* fat cells. (A–F) Projection images representing in TIRFM the number of trafficking HA-GLUT4-GFP particles in fat cells from transgenic larvae under basal conditions (A, C, E) or after insulin addition (B, D, F). (A, B) Animals were grown on standard food; (C, D) starved for 24 h; or (E, F) grown on sugar-restricted food. To enhance clarity images were inverted so that HA-GLUT4-GFP particles appear dark on a light background. Scale bar, 2 µm. Note increased number of trafficking particles for sugar-restricted larvae. (G) Quantification of trafficking (number of trafficking particles/100 µm^2^/min) under basal conditions (light gray bars) or 5 min after insulin stimulation (dark gray bars). Fat body tissue was analyzed from at least three independent animals for each condition. Statistical significance: *** p<0.001. On standard food, the difference between basal and insulin trafficking was not statistically significant (p>0.05). The total number of trajectories counted for 4 regions (10×10 µm) for each cell used to generate data for starved (basal-48; insulin 16); sugar-restricted (basal-48, insulin 16); standard (basal-48; insulin 16).

TIRFM was used to quantify GLUT4 trafficking by measuring the amount of mobile GLUT4 in the vicinity of the plasma membrane (number of trafficking particles/100 µm^2^/min) Fig. 2A–F show projection images which map trafficking GLUT4 particles. Fig. 2G shows quantification of these recordings for fat bodies from larvae reared on different diets and under basal conditions or after insulin addition, as indicated.

Under basal conditions, low levels of trafficking were observed (Figs. 2A and 2 G ‘Standard’, light gray bar, 4.4±0.3 trafficking particles/100 µm^2^/min; mean ± SEM). Within 5 min of insulin addition, the total amount of mobile GLUT4 increased slightly, representing the arrival and tethering to the membrane in the TIRF zone (Fig. 2B and 2G, ‘Standard’, dark gray bar, average 4.9±0.5 trafficking particles/100 µm^2^/min). Projection images mapping the trafficking under basal versus insulin stimulation shows a slightly higher number of trafficking particles over basal conditions, (Fig. 2A vs. 2B) and increased number of short-range movements was observed. In contrast to *Drosophila* fat, TIRFM studies on rat primary white adipocytes showed that insulin stimulation decreased GLUT4 trafficking due to vesicle tethering to the membrane [5], [8]. In addition, in mammalian cells, long-range movements of GLUT4 occurred along microtubule network [8], while shorter range movements were seen in *Drosophila* fat.

The small response to insulin described above was surprising but could be explained by the intense feeding behavior of larvae resulting in saturation and/or down regulation of insulin-responsiveness prior to the *ex vivo* stimulation experiments. To test this, animals were starved for 24 h (see Experimental Procedures), and fat bodies were dissected and incubated in the absence or presence of insulin, as above. Within 5 min of insulin addition, an increase in GLUT4 appearance in the TIRF zone was observed (Fig. 2C vs. Fig. 2D, Fig. 2G, ‘Starved’, 3.3±0.2 trafficking particles/100 µm^2^/min vs. 5.8±0.4 4 trafficking particles/100 µm^2^/min).

To further investigate the role of diet in competence to respond to insulin, total sugar uptake during feeding was reduced by rearing animals, from the point of egg laying through the time of collection, on sugar-restricted food (Experimental Procedures). Basal levels of trafficking were comparable to those seen for animals reared on standard food or starved (Fig. 2G). Addition of insulin resulted in a large increase in HA-GLUT4-GFP trafficking in fat from animals reared on sugar-restricted diets (Fig. 2E vs. 2F, 2G ‘Sugar-Restricted’, 3.9±0.2 trafficking particles/100 µm^2^/min vs. 10.9±0.7 trafficking particles/100 µm^2^/min). This is clearly seen by following particle movements in basal versus stimulated cells (see videos S1 vs. S2).

**Figure 3:**
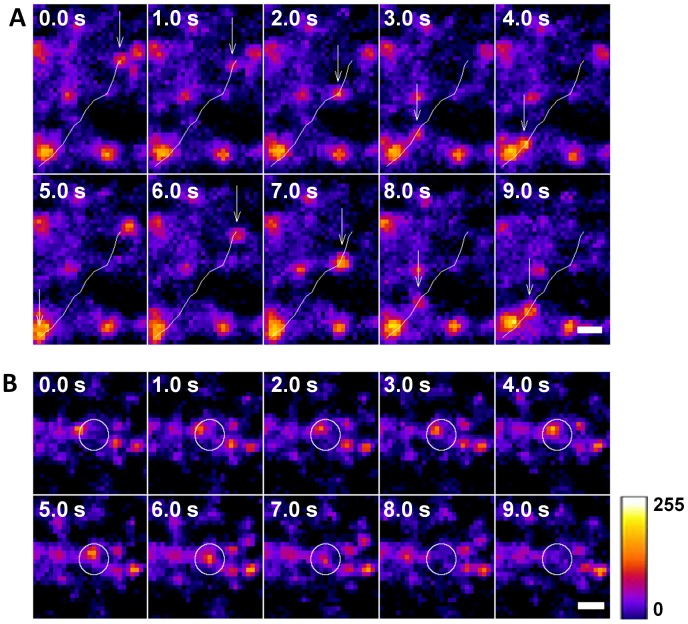
Trafficking, tethering and fusion of GLUT4 to the plasma membrane in *Drosophila* fat. (A) Time-lapse frames from TIRFM recording showing trafficking of GLUT4 in fat tissue isolated from sugar-restricted larvae. The white line indicates linear GLUT4 movement from one point to another, possibly along a microtubule network. Movement of two individual vesicle-like particles following the same trajectory is indicated by the white lines. The movement of the first particle is shown on the frames 0.0–5.0 s; the movement of the second particle is presented on the frames 6.0–9.0 s. Arrows indicate positions of the particles. (B) Time-lapse frames from TIRFM recording showing tethering and fusion of HA-GLUT4-GFP. White circles indicate the final position where tethering (1–7) and fusion takes place (8–10). Fluorescence intensity is shown in pseudocolor. Scale bars, 1 µm.

Time-lapse frames of TIRFM recordings showed linear movements of HA-GLUT4-GFP following insulin stimulation (Fig. 3A, arrows and white lines show the linear paths followed by two individual particles, 0.0 s–4.0 s and 5.0 s–9.0 s), reminiscent of microtubule-based trafficking seen in mammalian cells [8]. Note that trafficking of lipid droplets has also been observed by others in *Drosophila* tissues (reviewed [41]). The addition of insulin also induced the appearance of GLUT4 at the plasma membrane and tethering (one such vesicle fusion event is shown in Fig. 3B, white circles in panels 1–7), followed by fusion with the membrane, indicated by decreased fluorescence (Fig. 3B, white circles in panels, 8–10).

### Drosophila Harbor Machinery to Mediate GLUT4 Membrane Translocation in Response to Insulin

**Figure 4:**
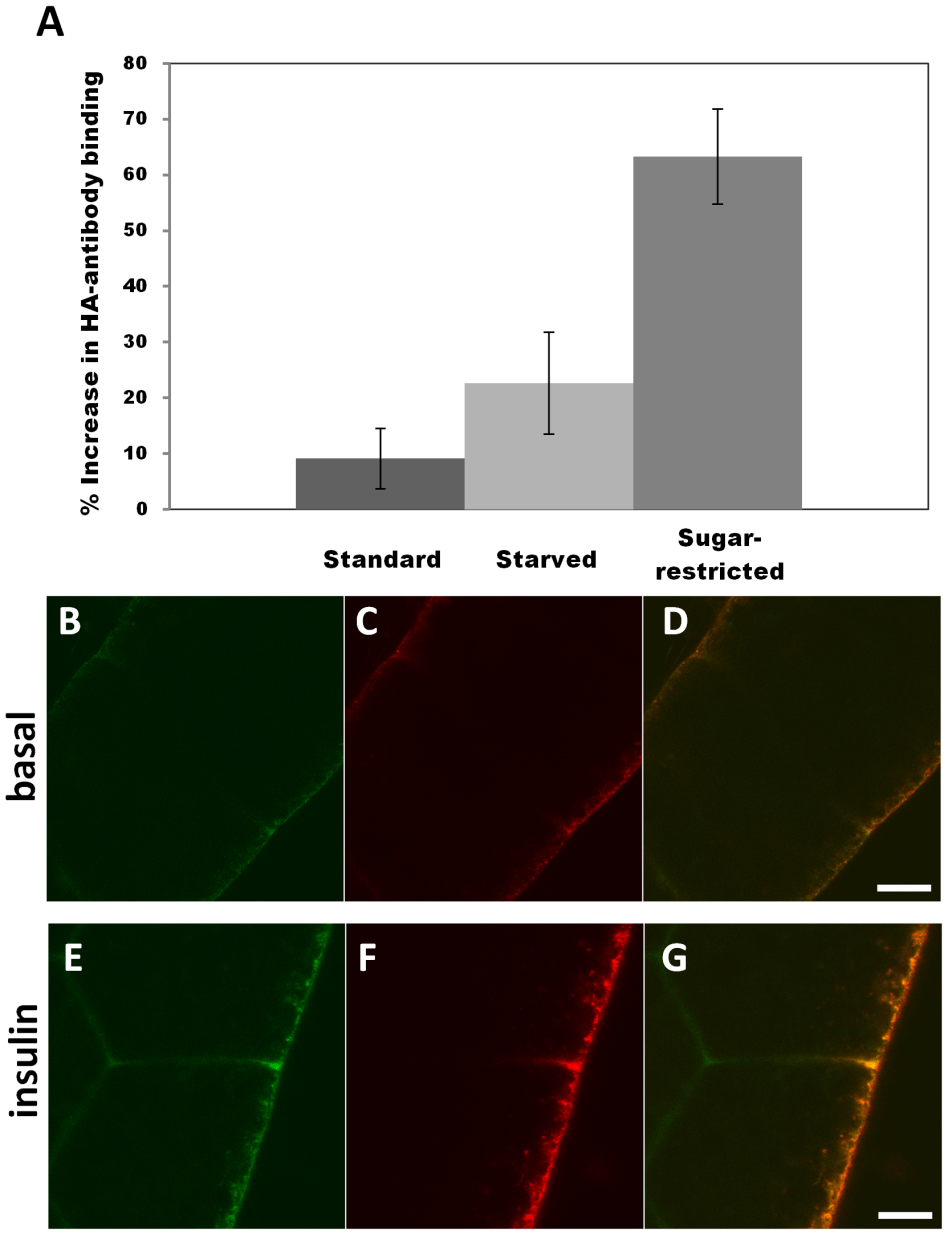
Insulin-stimulated GLUT4 translocation in *Drosophila* fat is enhanced by sugar restriction. (A) Surface exposure of HA-GLUT4 upon insulin stimulation. Fat bodies were collected from larvae reared on different dietary regimes, as indicated. After immunostaining of non-permeabilized fat body cells, values were determined by measuring fluorescence and averaging calculations of corrected integrated density. Values are expressed as percent of HA-GLUT4 fluorescence of insulin stimulation over basal conditions. Fat bodies were collected from three animals for each dietary regime. (B–G) Confocal microscopy of HA-GLUT4-GFP-expression in fat body cells from animals reared on a sugar-restricted diet in the absence or presence of insulin. (B, C, D) Basal conditions; (E, F, G) after addition of 0.1 U/ml insulin. GLUT4 was visualized in non-permeabilized cells by GFP fluorescence (green, B, E); or with anti-HA antibody (red C, F) to monitor membrane translocation; merged images (D,G). Scale bar is 5 µm. Note increase in GLUT4 at the cell surface (red, F).

The HA tag inserted in the extracellular domain of HA-GLUT4-GFP is detectable in non-permeabilized cells only after translocation to the surface [37], [42]. To test whether HA-GLUT4-GFP inserts into the plasma membrane in response to insulin in *Drosophila*, larval fat bodies were treated with insulin, and anti-HA antibody was used to monitor GLUT4 localization in non-permeabilized cells (Fig. 4 and Experimental Procedures). Treatment with insulin resulted in membrane translocation of HA-GLUT4-GFP in fat cells of animals reared on standard food, starved, or reared on a sugar-restricted diet (Fig. 4A). Average increases in anti-HA labeling on the surface of the plasma membrane was after insulin addition were ∼9%±5.4 (Mean ± SD) for animals fed standard diet; ∼23%±9.2 for starved larvae; and ∼63%±8.5 for larvae grown on sugar-restricted food. The latter increase is similar in magnitude to that seen in rat primary adipose tissue using the same insulin stimulation conditions [8]. These results suggest that continuous exposure to high sugar during intense larval feeding under standard conditions causes down-regulation of insulin responsiveness, similar to insulin-resistance induced in mammalian adipocyte cells [43]–[46].

**Figure 5:**
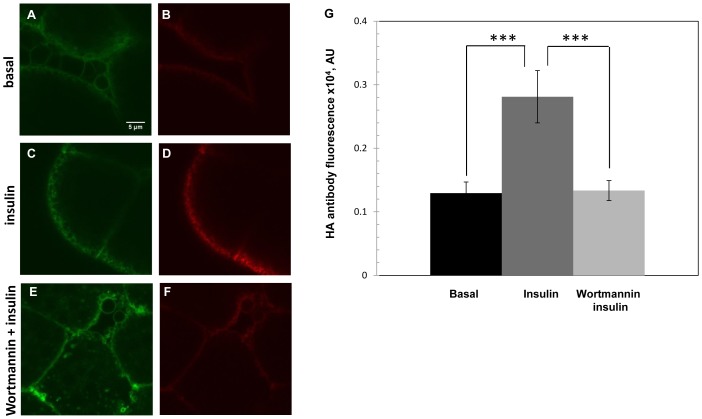
Insulin-stimulated GLUT4 translocation in *Drosophila* fat body is inhibited by wortmannin. Confocal microscopy of HA-GLUT4-GFP-expression in fat body cells from animals reared on sugar-restricted diets. (A, B) Basal conditions. (C, D) After addition of 0.1 U/ml insulin; or (E, F) After pretreatment with 100 nM/L wortmannin, followed by insulin addition. GLUT4 was visualized in non-permeabilized cells by GFP fluorescence (green, A, C, E) or with anti-HA antibody (red B, D, F) to monitor membrane translocation Scale bar, 5 µm. (G) Surface exposure of HA-GLUT4 upon wortmannin/insulin treatment (G, light gray) or insulin only (G, dark gray). Values are expressed as pixel intensity of HA-GLUT4 in various conditions, as indicated. Fat bodies were collected from five animals each. Anti-HA labeling showed a significant inhibition of the insulin response in wortmannin treated samples (F, G) where fluorescence intensity (AU, arbitrary units) (1335±157; mean±SEM) was comparable to basal samples (1294±175) (B, G).

Confocal images of fat bodies from animals reared on the sugar-restricted diet revealed similar levels of total GFP fluorescence under basal conditions (Fig. 4B) or after insulin stimulation (Fig. 4E), demonstrating that there was no change in the amount of total protein. However, staining with anti-HA antibody in these non-permeabilized cells revealed that insulin stimulated HA-GLUT4-GFP translocation to the membrane (compare Figs. 4C and F). The average increase in HA-labeling at the surface in these experiments (as well as those shown in Figure 5, see below) was approximately 2.5 fold, with a range of 2 to 4-fold in different samples. Note also that there was a high variation between cells in the total amount of HA-labeling due to different levels of expression of recombinant proteins in transgenic larvae. Thus, insulin stimulation of *Drosophila* larval fat cells triggers a change in the subcellular localization of HA-GLUT4-GFP similar to that seen in mammalian cells.

To test whether GLUT4 translocation to the membrane in response to human insulin engaged the endogenous *Drosophila* insulin signaling cascade, we utilized wortmannin, a known inhibitor of PI3 kinase activity [47], [48]. Fat bodies from larvae reared on sugar-restricted diets were treated with 100 nm/L wortmannin prior to the addition of insulin, as described above (see Experimental procedures) and translocation of HA-GLUT4-GFP to the membrane was observed by anti-HA staining of non-permeabilized cells, as above. Addition of insulin resulted in a significant increase in staining with anti-HA antibody in control non-wortmannin samples (Fig. 5 D vs. 5 B). In contrast, only a small increase over basal levels was observed in wortmannin treated samples (Fig. 5 F). The difference from basal levels was not statistically significant (p>0.05) (Fig. 5 G). As previously, overall levels of GFP fluorescence were not changed by insulin treatment (Fig. 5 A, C, and E). In conclusion, wortmannin blocked insulin stimulated translocation of GLUT4 to the plasma membrane, suggesting that insulin-induced GLUT4 translocation events observed in this study were supported by endogenous DInR/PI3K signaling pathways. However, it is worth noting that wortmannin also inhibits other kinases [49]–[51], which may impact the GLUT4 dynamics we observed.

## Discussion and Conclusions

We have shown that *Drosophila* harbor the machinery to mount an insulin-stimulated sugar uptake response. Fat cells isolated from transgenic *Drosophila* expressing human GLUT4 responded to insulin by increased trafficking and movement of GLUT4 to the plasma membrane. These responses were most clearly observed in animals reared on a sugar-restricted diet, suggesting that the same sugar transport pathways being measured in our *ex vivo* assays are functional in *Drosophila* larvae and are down-regulated during periods of high sugar uptake, mimicking mammalian insulin-resistance. In addition, this is, to our knowledge, the first study to use TIRFM to monitor sugar transporter trafficking in an insect, providing a useful new approach for the field to further dissect processes regulating sugar homeostasis.

Our studies of GLUT4 trafficking in *Drosophila* fat suggest that flies have transport mechanisms similar to mammals to regulate sugar uptake into cells. However, differences were also observed in TIRFM studies; for example, TIRFM revealed short-range movements in *Drosophila* fat as compared to long-range movement observed in primary adipose cells. We suggest that differences in the trafficking patterns and the amount of mobile GLUT4 seen in the vicinity of the plasma membrane (TIRF-zone) between mammalian white adipose cells and *Drosophila* fat cells can be explained by the drastically different organization of cytosolic space: in mammalian adipose cells, all vesicular trafficking takes place in the thin layer (∼0.5 µm) of cytosol surrounding the central lipid droplet, whereas in *Drosophila* fat cells, GLUT4 and other cytosolic structures are scattered among multiple smaller lipid droplets. Thus, while in mammalian adipose cells, GLUT4 vesicle trafficking takes place in the TIRF zone, in *Drosophila* fat cells, the majority of GLUT4 appears to move between lipid droplets and can be detected in the TIRF zone only after translocation to the vicinity of the plasma membrane. Consistent with this, the shorter range of movements observed in *Drosophila* fat are likely due to the high level of compartmentalization of the cytoplasm by fat droplets, which may result in fragmentation of the microtubular network. The dynamics of trafficking of GLUT4 particles before and after insulin stimulation in *Drosophila* appears to be more similar to that described for 3T3-L1 adipocytes than for primary adipose cells. Previous studies demonstrated that microtubules support GLUT4 trafficking and long-range movements along an intact microtubule network in primary adipose cells [8] and references therein). In 3T3-L1 adipocytes, multiple lipid droplets appear to fragment and shorten the cytoskeleton network [40]. This in turn is thought to be responsible for short-range movements of GLUT4 observed in these cells [8], [40]. In fly fat, we observed multiple lipid droplets, similar to what was seen in 3T3-L1 adipocytes, which may also fragment the cytoskeleton network in *Drosophila* fat, explaining the short-range movements we observed. In addition, TIRFM studies on rat primary white adipocytes showed that insulin stimulation decreased GLUT4 trafficking as a result of vesicle tethering to the membrane [5], [8]. In contrast, in 3T3-L1 adipocytes, insulin addition resulted in increased GLUT4 trafficking ([40], [52] and references therein), again similar to what we observed in *Drosophila* fat. Future studies will determine whether additional mechanistic differences in transporter homeostasis between mammals and flies also contribute to differences in trafficking patterns in mammals and insects.

In mammals, GLUT4 is stored in specialized vesicles, GLUT4 Storage Vesicles (GSVs), which upon insulin stimulation undergo translocation to the plasma membrane, that includes micro-tubule based trafficking, tethering to the membrane, and fusion, which concludes delivery of GLUT4 molecules to the cell surface (reviewed in [53]–[55]). GLUT4 packaging into GSVs is thought to be dependent on interaction with insulin-regulated aminopeptidase (IRAP), another resident protein of GSV [56]. Insulin stimulation changes the phosphorylation state of Rab GTPase-activating proteins TBC1D4/AS160 to induce tethering and fusion of the GSVs vesicles with plasma membrane [57]. While there may be an ortholog of AS160 in *Drosophila* [57], no homolog of IRAP has been identified in *Drosophila* to date; this absence of an IRAP-like molecule may explain the differences in trafficking we observed in *Drosophila* fat and that seen in mammalian cells. Thus, while our studies suggest that GLUT4 is present in vesicles in *Drosophila* fat, as indicated by the punctate structures seen in TIFRM (Fig. 1I, J, K), future work will determine whether GLUT4, but more importantly, endogenous *Drosophila* sugar transporters, localize to GSV-type vesicles and whether intracellular trafficking mechanisms are similar to those seen in mammals.

While mammals have nine insulin/insulin-like growth factor family members [58] the *Drosophila* genome encodes eight insulin-like peptides (DILPs), several of which are co-expressed in neurosecretory cells in the brain, termed Insulin Producing Cells (IPCs) [16], [59]. These *dilps* appear to be partially redundant in function, with at least two being regulated by nutritional status [10], [16]. Ablation of IPCs resulted in ‘diabetic’ flies with elevated circulating sugar levels [59]. Evidence that this rise in sugar levels in *Drosophila* was due to loss of DILP function came from studies showing that deletion of *dilp* genes resulted in small, developmentally delayed animals with decreased fat, slowed metabolic activity and elevated circulating trehalose [26], [60]. In our previous studies [26], in keeping with previous reports [59], [61], trehalose was the predominant circulating blood sugar in both wild type and *dilp* mutant flies, with free glucose accounting for only ∼2% of total sugar [25]. Additionally, injections of purified DILP5 in *Drosophila* resulted in a decrease in circulating trehalose [62]. DILPs may also be responsible for regulating hemolymph glucose, as IPC ablation increased circulating levels and direct injection of insulin decreased circulating glucose levels in *Drosophila* [63]. A role for the insulin receptor in this process was supported by RNAi knockdown of DInR in the fat body, which resulted in increased circulating sugar [64]. Similarly, Pasco and Leopold found that glucose levels increased after high sugar intake, and then stabilized at this increased level while trehalose levels remained constant, suggesting homeostatic regulation of both sugars [65]. These studies indicated that insulin-signaling directly controls levels of circulating sugar, although they did not address the mechanism regulating sugar levels. They further suggested that one or more DILPs function to decrease levels of circulating sugar in flies, reminiscent of insulin control of circulating sugar levels in mammals.

Here, we have added to this growing body of literature by showing that *Drosophila* fat cells respond to insulin by mounting a response similar to mammals, translocating the GLUT4 transporter to the cell membrane. This suggests that *Drosophila* fat cells respond to endogenous insulin-like signals by taking up sugar from circulation and that the ‘diabetic’ phenotypes seen in flies lacking DILPs results from failure of a DILP-stimulated sugar uptake response in fat and possibly other tissues. Together, these studies make a strong case for hormonal regulation of sugar homeostasis in insects and suggest that insulin-like signaling pathways are involved. Of prime interest will be identification of the endogenous sugar transporters mediating this insulin-dependent response in flies. The *Drosophila* genome harbors four candidate sugar transporters, two candidate trehalose transporters (Tret1–1 and Tret1–2) [66] and two candidate glucose transporters (Dmglut1 and Dmglut3) [67]. Future work will determine whether these transporter(s) are responsible for sugar homeostasis in insects and how they are regulated by endogenous DILP-signaling.

## Supporting Information

Figure S1 Expression and localization of HA-GLUT4-GFP in *Drosophila* fat body in two independent transformant lines (10C1 and 78A3). Fluorescence microscopy images confirmed HA-GLUT4-GFP-expression in fat body cells from animals reared on standard food. Virgin females homozygous for the *GAL4* driver were crossed to 10C1 or 78A3 transformant males with *UAS-HA-GLUT4-GFP*. Scale bar is 10 µm.
(TIF)

Video S1 **GLUT4 trafficking in** ***Drosophila*** **fat cells in basal conditions in animals reared on sugar-restricted diets.** Video shows 4x magnified regions of HA-GLUT4-GFP trafficking near the plasma membrane. Note that in basal conditions, little movement of vesicle-like particles was observed with the majority of particles being static, consistent with data shown in Figure 2. ovies 60 s time-lapse frames acquired using TIRF at the frame rate of 1 s is shown.
(AVI)

Video S2 **GLUT4 trafficking in** ***Drosophila*** **fat cells after insulin stimulation in animals reared on sugar-restricted diets.** Video shows 4x magnified regions of HA-GLUT4-GFP trafficking near the plasma membrane. Insulin resulted in an increase in the number of mobile GLUT4 particles and longer trajectories near the plasma membrane. Movie shows 60 s time-lapse frames acquired using TIRF at the frame rate of 1 s.
(AVI)
