# Supplementary figures and images for: Insulin Stimulates Translocation of Human GLUT4 to the Membrane in Fat Bodies of Transgenic Drosophila melanogaster
Source: PLoS One. 2013 Nov 6;8(11):e77953. doi: 10.1371/journal.pone.0077953 (PMC3819322; doi:10.1371/journal.pone.0077953)

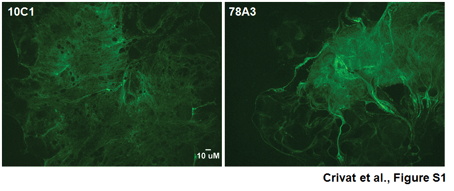

Supplement: Figure S1 — Expression and localization of HA-GLUT4-GFP in Drosophila fat body in two independent transformant lines (10C1 and 78A3). Fluorescence microscopy images confirmed HA-GLUT4-GFP-expression in fat body cells from animals reared on standard food. Virgin females homozygous for the GAL4 driver were crossed to 10C1 or 78A3 transformant males with UAS-HA-GLUT4-GFP. Scale bar is 10 µm. (TIF) [file pone.0077953.s001.tif]
